# Thymus Vulgaris Oil Nanoemulsion: Synthesis, Characterization, Antimicrobial and Anticancer Activities

**DOI:** 10.3390/molecules28196910

**Published:** 2023-10-02

**Authors:** Ahmed S. Doghish, Amr M. Shehabeldine, Hesham A. El-Mahdy, Mahmoud M. H. Hassanin, Abdulaziz A. Al-Askar, Samy A. Marey, Hamada AbdElgawad, Amr H. Hashem

**Affiliations:** 1 Department of Biochemistry, Faculty of Pharmacy, Badr University in Cairo (BUC), Cairo 11829, Egypt; ahmed_doghish@azhar.edu.eg; 2 Biochemistry and Molecular Biology Department, Faculty of Pharmacy (Boys), Al-Azhar University, Nasr City 11231, Egypt; heshamabbas@azhar.edu.eg; 3 Botany and Microbiology Department, Faculty of Science, Al-Azhar University, Nasr City 11884, Egypt; 4 Ornamental, Medicinal and Aromatic Plant Disease Department, Plant Pathology Research Institute, Agricultural Research Center (ARC), Giza 12619, Egypt; dr.hassanin.1978@gmail.com; 5 Department of Botany and Microbiology, Faculty of Science, King Saud University, P.O. Box 2455, Riyadh 11451, Saudi Arabia; aalaskara@ksu.edu.sa (A.A.A.-A.); samarey@ksu.edu.sa (S.A.M.); 6 Integrated Molecular Plant Physiology Research, Department of Biology, University of Antwerp, 2020 Antwerp, Belgium; hamada.abdelgawad@uantwerpen.be

**Keywords:** essential oil, nanoemulsion, *Thymus vulgaris* L., antimicrobial activity, anticancer activity, apoptosis

## Abstract

Essential oil nanoemulsions have received much attention due to their biological activities. Thus, a thyme essential oil nanoemulsion (Th-nanoemulsion) was prepared using a safe and eco-friendly method. DLS and TEM were used to characterize the prepared Th-nanoemulsion. Our findings showed that the nanoemulsion was spherical and ranged in size from 20 to 55.2 nm. The micro-broth dilution experiment was used to evaluate the in vitro antibacterial activity of a Th-emulsion and the Th-nanoemulsion. The MIC50 values of the thymol nanoemulsion were 62.5 mg/mL against *Escherichia coli* and *Klebsiella oxytoca*, 250 mg/mL against *Bacillus cereus*, and 125 mg/mL against *Staphylococcus aureus.* Meanwhile, it emerged that the MIC50 values of thymol against four strains were not detected. Moreover, the Th-nanoemulsion exhibited promising antifungal activity toward *A. brasiliensis* and *A. fumigatus*, where inhibition zones and MIC50 were 20.5 ± 1.32 and 26.4 ± 1.34 mm, and 12.5 and 6.25 mg/mL, respectively. On the other hand, the Th-nanoemulsion displayed weak antifungal activity toward *C. albicans* where the inhibition zone was 12.0 ± 0.90 and MIC was 50 mg/mL. Also, the Th-emulsion exhibited antifungal activity, but lower than that of the Th-nanoemulsion, toward all the tested fungal strains, where MIC was in the range of 12.5–50 mg/mL. The in vitro anticancer effects of Taxol, Th-emulsion, and Th-nanoemulsion were evaluated using the standard MTT method against breast cancer (MCF-7) and hepatocellular carcinoma (HepG2). Additionally, the concentration of VEGFR-2 was measured, and the activities of caspase-8 (casp-8) and caspase-9 (casp-9) were evaluated. The cytotoxic effect was the most potent against the MCF-7 breast cancer cell line after the Th-nanoemulsion treatment (20.1 ± 0.85 µg/mL), and was 125.1 ± 5.29 µg/mL after the Th-emulsion treatment. The lowest half-maximal inhibitory concentration (IC50) value, 20.1 ± 0.85 µg/mL, was achieved when the MCF-7 cell line was treated with the Th-nanoemulsion. In addition, Th-nanoemulsion treatments on MCF-7 cells led to the highest elevations in casp-8 and casp-9 activities (0.66 ± 0.042 ng/mL and 17.8 ± 0.39 pg/mL, respectively) compared to those with Th-emulsion treatments. In comparison to that with the Th-emulsion (0.982 0.017 ng/mL), the VEGFR-2 concentration was lower with the Th-nanoemulsion treatment (0.672 ± 0.019ng/mL). In conclusion, the Th-nanoemulsion was successfully prepared and appeared in nanoform with a spherical shape according to DLS and TEM, and also exhibited antibacterial, antifungal, as well as anticancer activities.

## 1. Introduction

The advantages associated with plant compounds have been known since time immemorial. Their advantages, owing to their diverse medicinal characteristics, are becoming better understood [1]. Several investigations have shown that they have increasing uses as antioxidants, antimicrobials, anti-tumor and anti-inflammatory agents, and immunological modulators. In certain circumstances, they may be alternative substitutes for antibiotics [2]. The abuse of antimicrobial medicines has resulted in the development and spread of bacterial species resistant to medications [3]. The prevalence of multidrug-resistant (MDR) bacteria is increasing, highlighting the growing worry regarding effective treatment choices for illnesses triggered by these pathogens [4]. Due to the lack of effective treatments, substantial instances of morbidity and death have been seen in humans who have been exposed to microorganisms that are extensively and/or multidrug-resistant (MDR) [5]. The surprising failure of therapy has been caused by the widespread proliferation of MDR pathogens [6,7]. MDR pathogens are troublesome notwithstanding the creation of innovative medications due to the sharp rise in the number of affected patients and the bacterial adoption of genomic susceptibility factors [8]. As a result, microbial resistance is an important issue for the community, and there is a need to research and identify novel chemicals with antimicrobial activities that have no adverse effects on the host body [9]. Cancer is another problem due to its high incidence rate; cancer is the second primary cause of death worldwide and has become a global health concern in the twenty-first century. Annually, there are approximately 15 million death cases due to the persistence of malignant cells, and the number of cases is steadily rising [10]. Currently, there are many chemotherapeutic drugs for treating cancers, but they cause serious side effects on most human organs [11]. Cancer treatment has always been a very difficult process. Although conventional therapies including surgery, chemotherapy, and radiotherapy have been employed, major strides have lately been made with the use of stem cell therapy and microRNA-targeted therapy, radionics, chemodynamic therapy, sonodynamic therapy, nanoparticles, natural antioxidants, ablation therapy, and therapy based on ferroptosis [12].

Essential oils (EOs) as natural antibacterial agents have gained increasing attention in the last decade. EOs are recognized as naturally occurring bioactive substances that can be used to prevent the spread of infections. However, because of the high volatility of their certain elements, the direct inclusion of EOs poses technical difficulties [13]. These active compounds have demonstrated significant antibacterial and antifungal activity. They are effective against a wide range of bacteria, including those involved in medically relevant diseases [14]. The most volatile component of thyme essential oil is thymol [15]. Thymol is generally believed to be safe, and it has been used as an antibacterial and antioxidant compound [16]. However, thymol’s drawbacks that restrict its utilization include volatility, poor stability, and high hydrophobicity [17].

The formation of nanoemulsions of thyme essential oil provides an appropriate and successful method for improving the structural integrity of the active components and their bioactivity [18]. The technology of nanoemulsion production provides several advantages with respect to the dispersed active principle and formulation stability, such as (i) better protection of the active agent against chemical or biological degradation, (ii) lower probability of creaming or sedimentation of droplets, (iii) greater contact surface of the target with the droplets that contain the active agent, (iv) possibility of dispersion of immiscible substances in a certain solvent, which in the case of EOs is usually water, besides the simplicity of production, (v) low cost of reagents, and (vi) less residual damage to the environment when compared to synthetic products, widely used in modern times [19,20,21].

A class of protein kinases known as receptor tyrosine kinases uses signal transduction pathways to regulate intra- and intercellular signaling. The control of cellular functions like growth, proliferation, differentiation, survival, and metabolism is significantly influenced by these proteins [22]. Of them, the receptor for vascular endothelial growth factor-2 (VEGFR-2) is an essential modulator of the migration and proliferation of endothelial cells [23]. VEGFR-2 has been identified as the primary driver of cancer cell proliferation, migration, and angiogenesis in the context of cancer cells [24]. Furthermore, scientific investigation has revealed that a notable overexpression of VEGFR-2 is found in a variety of cancer types. Given its critical role in the regulation of angiogenesis, VEGFR-2 thus poses a major therapeutic target for the inhibition of cancer growth and metastasis [25]. Herein, this study aimed to (1) prepare a stable and homogenous thyme essential oil nanoemulsion, (2) assess its antibacterial and antifungal activity toward pathogenic bacterial and fungal strains, and (3) evaluate the apoptosis markers casp-8 and casp-9 as well as the VEGFR-2 protein to determine their anticancer activity against MCF-7 and HepG2 cell lines.

## 2. Results and Discussion

### 2.1. Preparation and Characterization of Th-nanoemulsion

Thyme essential oil was used for the preparation of the Th-nanoemulsion using the ultrasonication method [26]. The result revealed that the color changing to white indicated the formation of an emulsion or nanoemulsion according to the used method. To confirm the formation of the Th-nanoemulsion, dynamic light scattering and TEM analyses were carried out.

**Figure 1:**
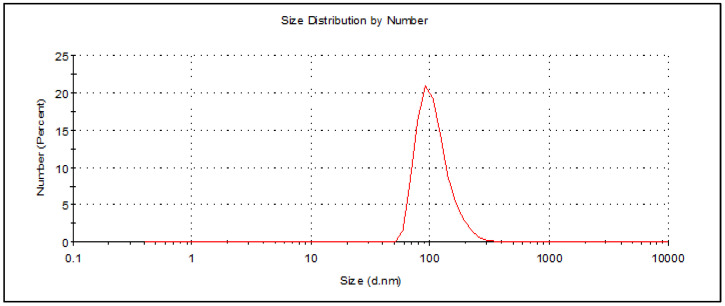
Particle size of the Th-nanoemulsion prepared by an ultrasonication method for 20 min. Peak at 91.28 nm and PDI = 0.272.

The technique of dynamic light scattering (DLS) is employed to assess colloidal stability by quantifying the size and distribution of particles or droplets. In addition, droplet size is significantly influenced by cavitation, turbulence, and shear forces generated by the ultrasonic homogenizer. Furthermore, the type of emulsifiers employed can also have an impact due to their ability to reduce the interfacial tension between the dispersed and continuous phases [27]. Figure 1 depicts a stabilized Th-nanoemulsion that was prepared using the ultrasonication method for 20 min at a power output of 350 W. The nanoemulsion was then stored at room temperature for 10 days. Tween 80, a non-ionic surfactant with a high hydrophilic–lipophilic balance (HLB) value, was employed in this study to facilitate the formulation of oil-in-water emulsions. The analysis demonstrated that the dimensions of the Th-nanoemulsion were measured to be approximately 91.28 nm. The reduction in particle size is contingent upon the efficacy and functionality of the surfactant, with Tween 80 being employed as a surfactant possessing a high hydrophilic–lipophilic balance (HLB) value, thereby promoting the formulation of oil-in-water emulsions. The polydispersity index (PDI) serves as a metric for assessing the uniformity and consistency of the droplet size distribution in nanoemulsions. Typically, the polydispersity index (PDI) falls within the numerical interval of 0 to 1. The nanoemulsion system exhibited a narrow size distribution and high uniformity, as indicated by the PDI below 0.3. When the PDI exceeds a threshold of 0.4, the system exhibits a wide range of particle sizes, thereby increasing the likelihood of coalescence [28]. The Th-nanoemulsion exhibited uniform properties with a PDI of 0.272, thus confirming that it was stable and homogenous (Figure 1).

**Figure 2:**
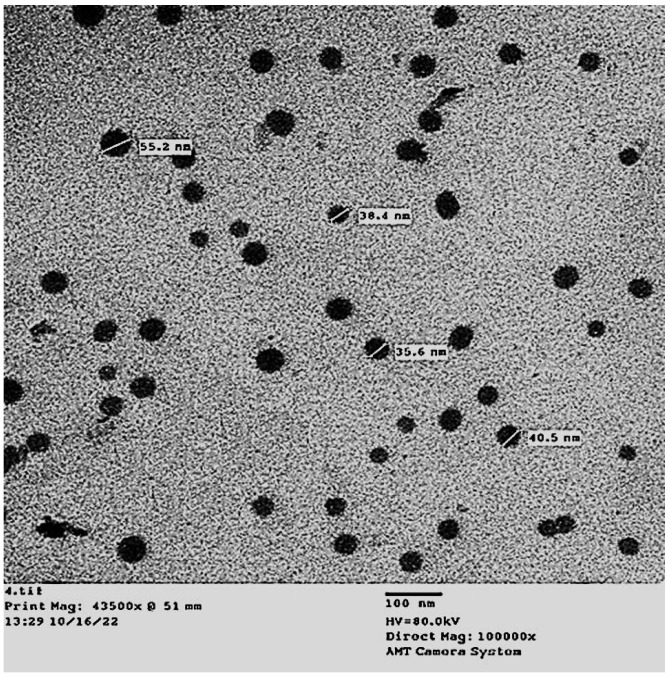
Transmission electron microscope image of thyme essential oil nanoemulsion prepared by an ultrasonication method for 20 min (size ranging from 91 to 182 nm).

The Th-nanoemulsion was subjected to transmission electron microscopy (TEM) analysis for characterization, revealing valuable insights into its size and shape. The resulting images provided an accurate representation of the dimensions and morphology of the nanoemulsion. Notably, the droplets within the nanoemulsion exhibited a distinct dark appearance, indicative of their presence and distribution within the sample. TEM micrographing showed that the nanoemulsion of essential oils was monodisperse spherical. Also, the size of the Th-nanoemulsion droplets was in the range of 20–55.2 nm (Figure 2). Previous studies have confirmed that most essential oil nanoemulsions appear with a spherical shape [29,30]. In a previous study, a Th-nanoemulsion has been prepared and characterized, and it was reported that the shape was spherical, with sizes of 164–252 nm [29]. Likewise, a Th-nanoemulsion has successfully been prepared and appeared spherical in shape with sizes of 40 to 110 nm [30]. Also, El-Sayed and El-Sayed [31] reported that a Th-nanoemulsion appeared spherical with size in the range of 30.4–52 nm. Likewise, Sundararajan, Moola [32] found that a prepared Th-nanoemulsion was spherical with a homogenous distribution.

### 2.2. In Vitro Antimicrobial Activity

**Figure 3:**
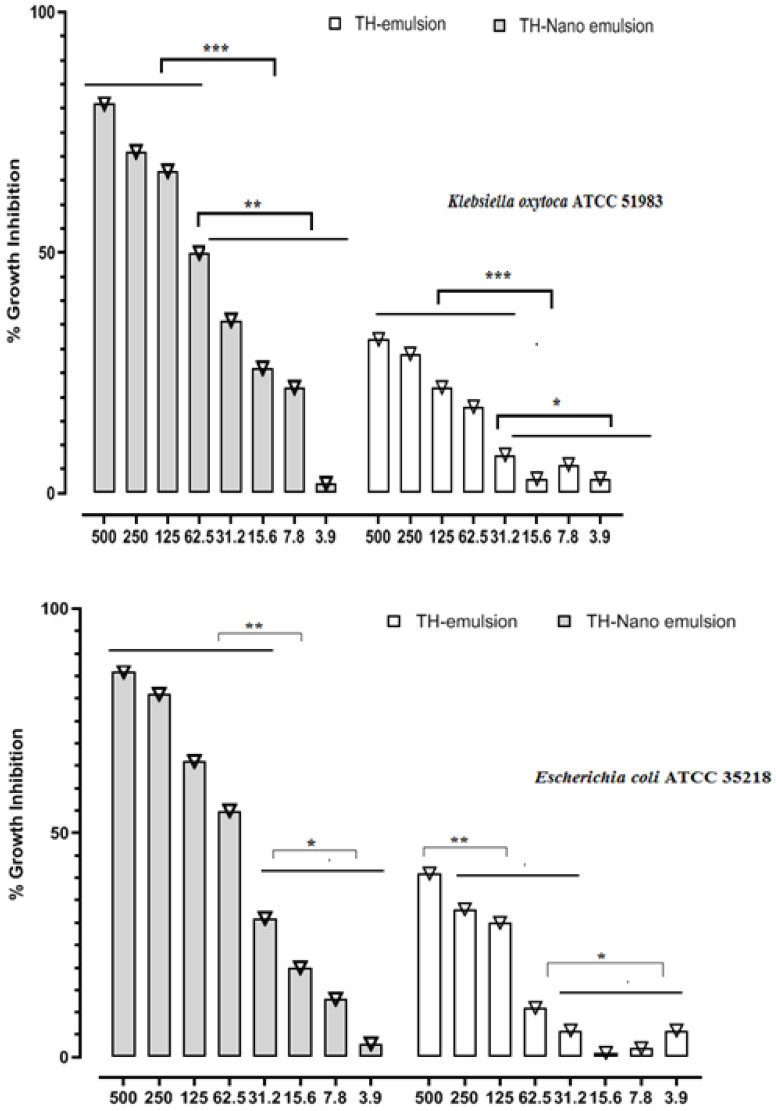

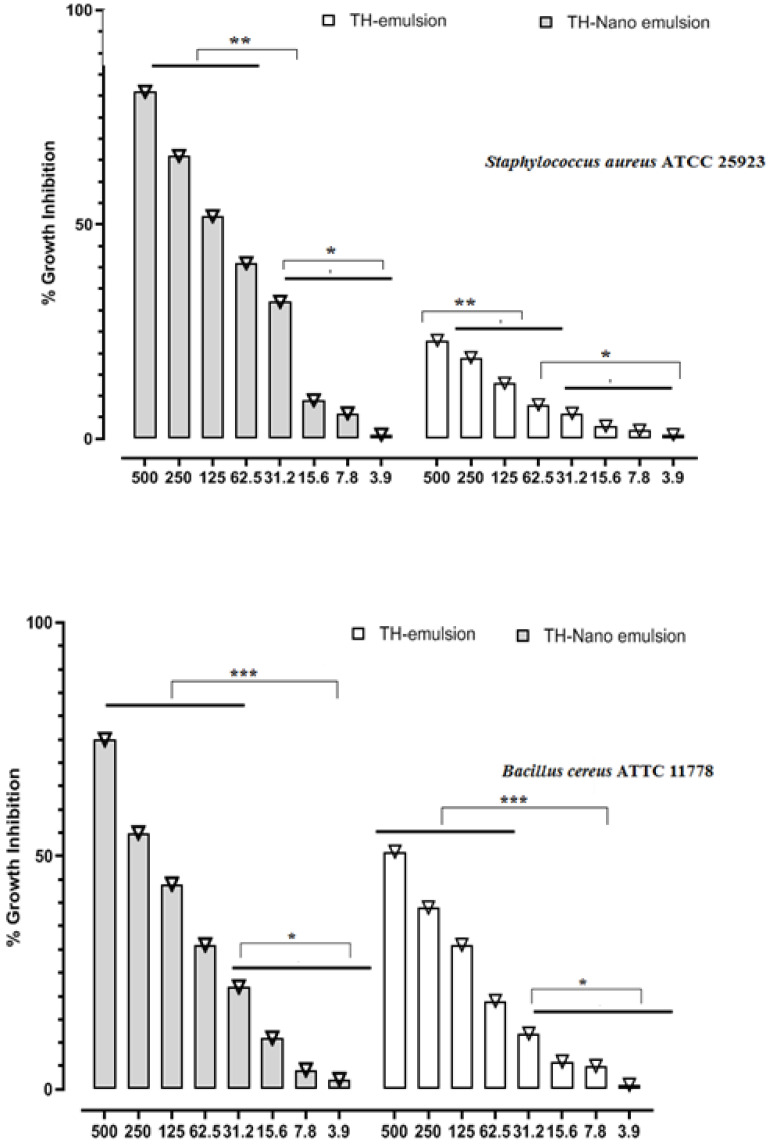
MIC50; 50% inhibition concentration (mg/mL) [37]. The Th-emulsion and Th-nanoemulsion limited the proliferation of bacteria in a concentration-dependent manner against *B. cereus* ATTC 11778, *S. aureus* ATCC 25923, *E. coli* ATCC 35218, and *K. oxytoca* ATCC 51983 at the indicated doses that were evaluated using a micro-broth dilution assay in a BHI broth. The percentage growth inhibition related to the vehicle (0.25% DMSO) is shown as the mean SE of three separate trials. One-way ANOVA was used for statistical analysis, and Tukey’s test was used to evaluate mean differences; * *p* < 0.05, ** *p* < 0.01, and *** *p* < 0.001.

The disc diffusion technique and micro-broth dilution test were used to determine preliminary levels of thymol emulsion and thymol nanoemulsion against *Bacillus cereus* ATTC 11778, *Staphylococcus aureus* ATCC 25923, *Escherichia coli* ATCC 35218, and *Klebsiella oxytoca* ATCC 51983. All the studied organisms were chosen because of their outstanding resistance to different antibiotics. Table 1 summarizes the sizes of the regions of inhibition. The antimicrobial effects of thymol and the nanoemulsion against microorganisms inspected within the display were subjectively and quantitatively evaluated by the nearness or nonappearance of a restraint zone distance across (DD) and medium inhibitory concentration (MIC50). The greatest quantity of thymol nanoemulsion at 50 mg/mL inhibited *B. cereus*, *S. aureus*, *E. coli*, and *K. oxytoca* with 25, 26, 23, and 21 mm inhibition zones, respectively. The agar disk dissemination test showed that ciprofloxacin was an essentially more grounded antimicrobial agent against the tried microorganisms, shaping the largest hindrance zone distances across (Table 1). The medium inhibitory concentration (MIC50) values of thymol and the nanoemulsion containing thymol are presented in Table 1 and Figure 3. According to Table 1, the MIC50 values of the thymol nanoemulsion were 62.5 mg/mL against *E. coli* and *K. oxytoca*, 250 mg/mL against *B. cereus,* and 125 mg/mL against *S. aureus*, respectively. Meanwhile, it emerged that the MIC50 values of thymol against four strains were not detected. Critical antibacterial movement of the thymol nanoemulsion was taken note of at a really low concentration, which was irrefutably much superior to that of the thymol emulsion. The MIC50 and MBC of the thymol nanoemulsion were much lower than those of thymol against all the tested bacteria (Table 1). In this regard, the MIC50 and MBC values for thymol were around three times those of the thymol nanoemulsion, showing that the nanoemulsion was more efficient in preventing the growth of the tested organisms. The capacity of thymol to disrupt the lipid component of bacterial membranes may be related to its antibacterial properties [33]. Thymol has a phenolic hydroxyl on its phenolic ring, which improves its hydrophilicity and allows it to disintegrate in antimicrobial membranes without damaging them [34]. As a result, thymol enhances permeation of the membrane, also decreasing the bilayer equilibrium, leading to internal material loss. Thymol disrupts membrane integrity and increases membrane permeability, resulting in cellular potential loss [35]. The hydroxyl group on thymol is crucial for depolarizing membrane potential and lowering the membrane potential. Additionally, thymol may affect the DNA secondary structure, altering the shape of DNA [36]. The antibacterial activity of the thymol nanoemulsion was ascribed to its nanoscale dimension and increased surface area, which allowed thymol to get inside and damage cellular membranes, resulting in the contents of the cells spilling.

### 2.3. Antifungal Activity

**Figure 4:**
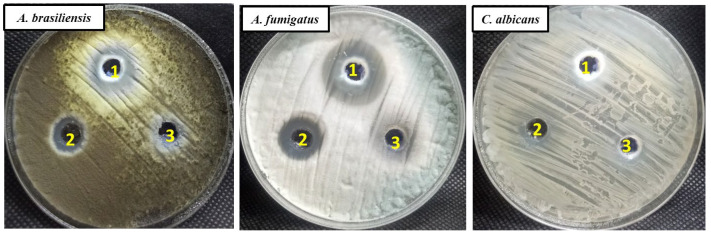
Antifungal activity of the Th-emulsion and Th-nanoemulsion toward *A. brasiliensis*, *A. fumigatus*, and *C. albicans* using the agar well diffusion method. Numbers 1, 2, and 3 mean Th-nanoemulsion, Th-emulsion, and nystatin, respectively.

Nanoemulsions have received much attention for biomedical and agricultural applications recently. In the current study, the prepared Th-emulsion and Th-nanoemulsion were assessed for antifungal activity toward *A. brasiliensis*, *A. fumigatus*, and *C. albicans* as illustrated in Figure 4. The Th-nanoemulsion displayed promising antifungal activity against filamentous fungi more than against unicellular fungi. Furthermore, the Th-nanoemulsion exhibited outstanding antifungal activity toward *A. brasiliensis* and *A. fumigatus*, where inhibition zones at a concentration of 100 mg/mL were 20.5 ± 1.32 and 26.4 ± 1.34 mm, respectively, but weak antifungal activity against *C. albicans* with an inhibition zone 12.0 ± 0.90 mm. Moreover, the Th-emulsion exhibited antifungal activity lower than that of the Th-nanoemulsion, with inhibition zones of 13.2 ± 1.41, 18.3 ± 1.1, and 12.7 ± 0.88 mm against *A. brasiliensis*, *A. fumigatus*, and *C. albicans*, respectively.

Also, the MICs and MFCs of the Th-emulsion and Th-nanoemulsion toward all fungal strains were detected. The MICs and MFCs of the Th-nanoemulsion against *A. brasiliensis*, *A. fumigatus*, and *C. albicans* were 12.5, 6.25, and 50; and 50, 12.5, and 100 mg/mL, respectively (Table 2), where *A. fumigatus* was the most sensitive and *C. albicans* was the most resistant. Additionally, the MICs and MFCs of the Th-emulsion toward *A. brasiliensis*, *A. fumigatus*, and *C. albicans* were 50, 12.5, and 50; and 100, 50, and 100 mg/mL, respectively (Table 2).

Several bioactive substances found in thyme oil, including thymol and carvacrol, have been proven to have antifungal effects [38]. These substances can damage the fungal cell membrane, causing the contents of the cell to flow out and cell death. The formulation of the nanoemulsion aids in the more efficient delivery of these substances to the fungal cells, increasing their antifungal effectiveness [39]. Moreover, thyme oil and its bioactive compounds can induce the generation of reactive oxygen species (ROS) within fungal cells. ROS, such as hydrogen peroxide and superoxide radicals, may damage biological components such as proteins, lipids, and DNA. This oxidative stress has the potential to impair essential cellular processes, eventually leading to fungal cell death [40]. Also, the nanoemulsion’s tiny droplet size enhances the surface area of thyme oil, allowing for greater interaction with fungal cells. This increased surface area improves the interaction of thyme oil with the fungal cell membrane, resulting in better antifungal activity [41]. Moreover, thyme oil has been demonstrated to inhibit a variety of enzymes required for fungus growth and survival. It may, for example, block enzymes involved in energy metabolism, such as ATP synthase, resulting in reduced ATP synthesis and consequent disruption of cellular functions in fungi [42].

### 2.4. Cytotoxic Effect of Th-emulsion and Th-nanoemulsion on MCF-7 and HepG2 Cancer Cell Lines

**Figure 5:**
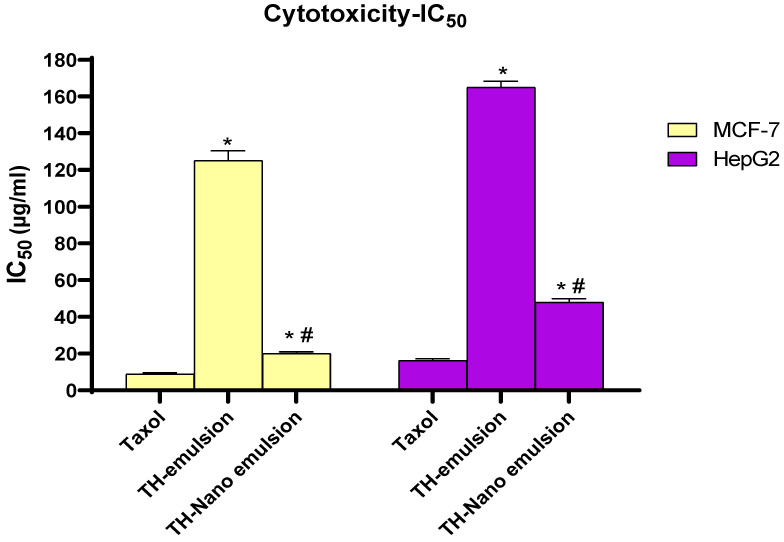
Effect of Taxol, Th-emulsion, and Th-nanoemulsion on MCF-7 and HepG2 cancer cell lines. The results are reported as the mean ± SD of three separate tests. * Significant *p*-values from the Taxol group at *p* < 0.001, # significant *p*-value from the Th-emulsion group at *p* < 0.001.

The cytotoxic activity of the Th-emulsion and Th-nanoemulsion was examined on two different cancer cell lines, MCF-7 and HepG2. As shown in Figure 5, MCF-7 breast cancer cells had the lowest half-maximal inhibitory concentration (IC50) values, indicating the most effective cytotoxic impact. The lowest MMIC50 value (20.1 ± 0.85 µg/mL) was obtained after Th-nanoemulsion treatment of the MCF-7 cell line. Furthermore, Th-emulsion treatment of the MCF-7 cell line produced an IC50 value of 125.1 ± 5.29 µg/mL. Taxol’s IC50 was 8.9 ± 0.73 µg/mL.

A common chemotherapy drug used to treat various malignancies is Taxol, sometimes referred to as paclitaxel. Over a million patients have been treated with Taxol since its antitumoral activity was discovered, making it one of the most commonly used antitumoral medications. With its primary mode of action being the disruption of microtubule dynamics, which results in mitotic arrest and cell death, Taxol was the first microtubule-targeting drug to be reported in the literature. Nevertheless, secondary pathways have also been shown for apoptosis that involve the elevation of reactive oxygen species (ROS) levels and the upregulation of genes and proteins associated with endoplasmic reticulum (ER) stress. Nevertheless, further investigation is required to determine whether the strain on the endoplasmic reticulum is a result of gene dysregulation induced by p53 activation. Conversely, there exists a proposition that impairment of the endoplasmic reticulum (ER) may induce the liberation of calcium ions (Ca2+), hence instigating an excessive accumulation of Ca2+ and subsequent mitochondrial impairment. This cascade of events ultimately results in an elevation of ROS generation [43].

Our results showed that the nanoemulsion on MCF-7 showed lower IC50 values than the emulsion form did, which is in agreement with the study that was performed by Tawfik, Teiama [44], who reported that the anticancer dosage of a thalidomide analog was significantly lowered from micromolar efficiency to nanomolar efficiency using the nanoemulsion formula [44]. Furthermore, our study concurs with another study that reported both pure essential oil of *Nigella sativa* and its two nanoemulsions showing antiproliferative efficacy against hepatocellular carcinoma cells that was dose-dependent. Under nanoemulsion conditions, its activity concerning cell inhibition increased [45].

### 2.5. Effect of Th-emulsion and Th-nanoemulsion on Caspase-8 and Caspase-9 Activities

**Figure 6:**
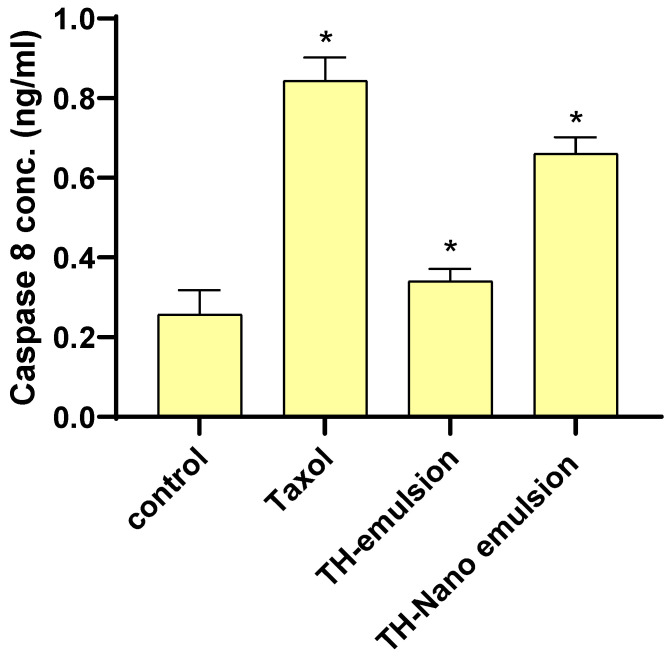
Effects of Taxol, Th-emulsion, and Th-nanoemulsion on caspase-8 in MCF-7 cells. The data are represented as the mean ± SD; * significant from the control group at *p*-value < 0.0001.

**Figure 7:**
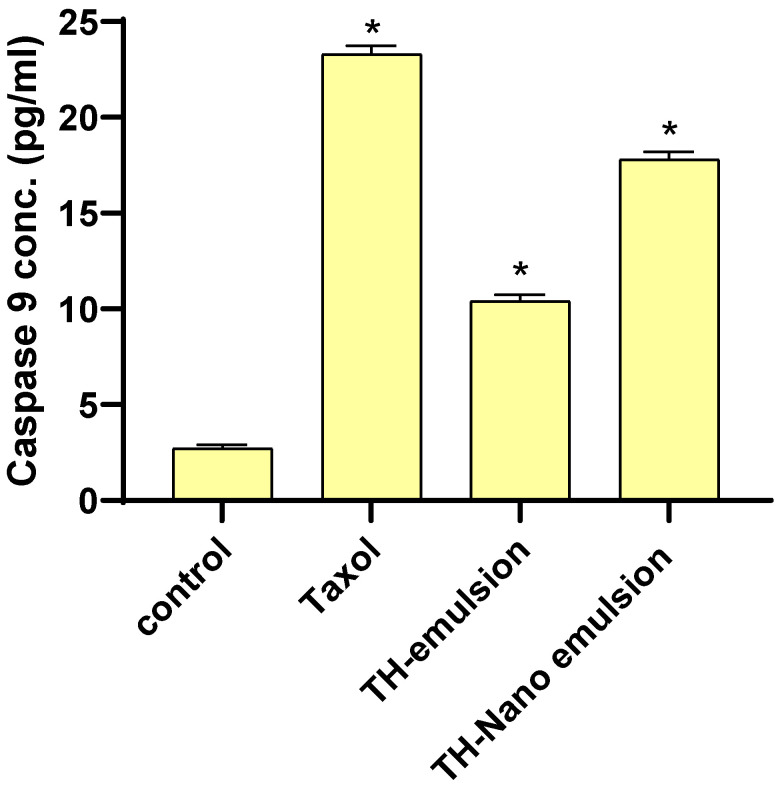
Effects of Taxol, Th-emulsion, and Th-nanoemulsion on caspase-9 in MCF-7 cells. The data are represented as the mean ± SD, *: significant from the control group at *p*-value < 0.0001.

The effects of the Th-emulsion and Th-nanoemulsion on the apoptosis markers casp-8 and casp-9 are described in Figure 6 and Figure 7. The activities of casp-8 and casp-9 were significantly increased in the treatment of MCF-7 cells with the Th-emulsion (0.34 ± 0.031 ng/mL and 10.4 ± 0.33 pg/mL, respectively) in comparison to those in the control (0.257 ± 0.061 ng/mL and 2.714 ± 0.19 pg/mL, respectively). Additionally, the treatment of MCF-7 cells with the Th-nanoemulsion achieved the highest increase in both casp-8 and 9 activities (0.66 ± 0.042 ng/mL and 17.8 ± 0.39 pg/mL, respectively) when compared with those in Th-emulsion treatments. Through the activation of casp-8 and 9, apoptosis promotes DNA fragmentation enzymes [46]. These substances activated casp-8 and casp-9, causing MCF-7 cells to undergo apoptosis.

### 2.6. Effect of Th-emulsion and Th-nanoemulsion on VEGFR-2

**Figure 8:**
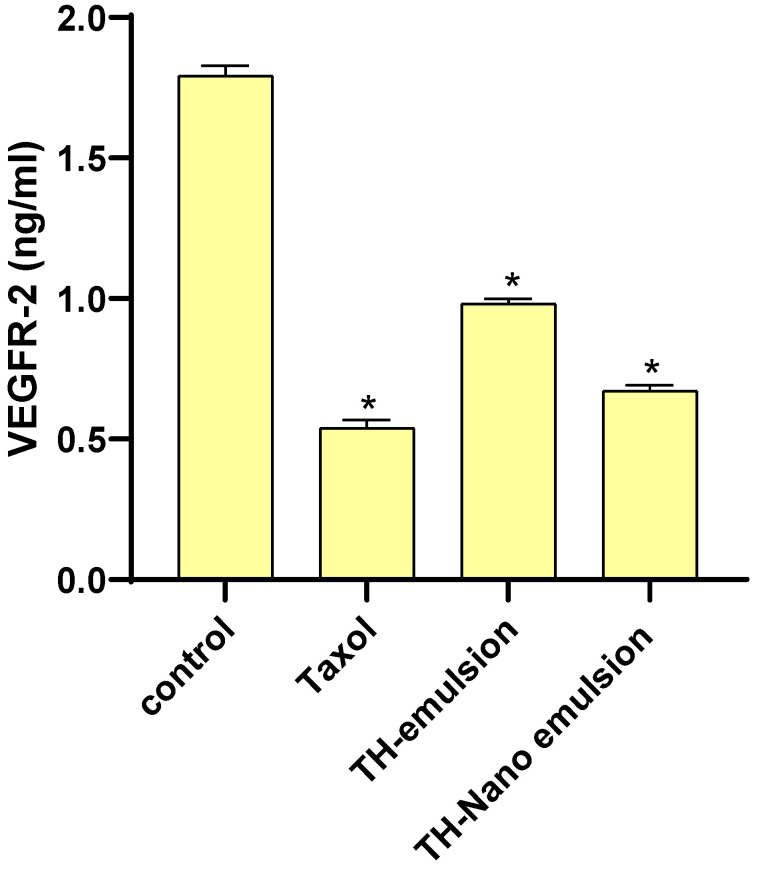
Effects of Taxol, Th-emulsion, and Th-nanoemulsion on VEGFR-2 in MCF-7 cells. The data are represented as the mean ± SD; * significant from the control group at *p*-value < 0.0001.

As represented in Figure 8, the Th-emulsion and Th-nanoemulsion significantly decreased the level of VEGFR-2 compared to that in the control (1.793 ± 0.036 ng/mL). The Th-nanoemulsion treatment gave a lower level of VEGFR-2 (0.672 ± 0.019 ng/mL) than the Th-emulsion did (0.982 ± 0.017 ng/mL). This result is in agreement with that of Falcon et al., who suggested that increased VEGFR-2 activity is a mediator of angiogenesis, which supports the development of solid tumors. Treatment for various cancer types, including breast cancer, now often includes inhibiting the VEGFR-2 pathway [47].

## 3. Materials and Methods

### 3.1. Chemicals and Reagents

Taxol (paclitaxel) was obtained from Sigma Chemical in St. Louis, MO, USA. 3-(4,5-Dimethyl-2-thiazolyl)-2,5-diphenyl-2H-tetrazolium bromide (MTT) and dimethyl sulfoxide (DMSO) were supplied by Sigma Aldrich (Sigma, St. Louis, MO, USA). Fetal bovine serum (FBS), phosphate buffer saline (PBS), Dulbecco’s modified Eagle’s medium (DMEM), penicillin/streptomycin (Pen/Strep) solution, and trypsin-EDTA were among the materials bought from Gibco (Gibco, TFS Inc., New York, NY, USA). HepG2 and MCF-7 were donated by ATCC (Manassas, VA, USA) and then grown in DMEM with 10% FBS and 1% Pen/Strep solution at 37 °C with 5% CO2.

### 3.2. Preparation of Nanoemulsion (NE)

Thyme essential oil was purchased from the Medicinal Plants Research Department at the Horticultural Research Institute, Agricultural Research Center in Giza, Egypt. A composite emulsifier was created by slowly adding 20 mL of essential oil and 5 mL of non-ionic surfactant Tween 80 with gentle stirring until a homogeneous mixture was formed. Subsequently, 75 mL of water was added to each oil sample, resulting in a final mixture volume of 100 mL. The mixture was then subjected to magnetic stirring for 15 min to ensure thorough homogenization. The mixture underwent sonication using an ultrasonicator (BANDELIN SONOPULS HD 220 ProfiLab24 GmbH, Berlin, Germany) for 20 min at a power output of 350 W. Throughout the process, the prepared essential oil nanoemulsion samples were kept in an ice bath. The particle size of the nanoemulsion containing 10% essential oil was measured using a hydrodynamic light scattering analyzer (DLS) following 10- and 90-day storage periods at room temperature (27 °C). The essential oil emulsion was prepared as previously described, without the use of sonication [48].

### 3.3. Measurement of Particle Size

Thyme essential oil was subjected to measurement using dynamic light scattering (DLS) analysis with a Zeta Nano ZS instrument (Malvern Instruments, Malvern, Worcestershire, UK) under ambient conditions. Before conducting the measurement, 30 μL of the nanoemulsion was diluted with 3 mL of water at a temperature of 25 °C. The particle size data was quantified by calculating the mean of the Z-average from three separate batches of the nanoemulsion. The size of the droplets and the polydispersity index (PDI) of the prepared nanoemulsion were determined.

### 3.4. Transmission Electron Microscopy (TEM)

A volume of 20 μL of the diluted sample was applied onto a copper sample grid coated with a 200-mesh film. The grid was allowed to incubate for 10 min, after which any excess liquid was eliminated using filter paper. Subsequently, a single droplet of a phosphotungstic acid solution with a concentration of 3% was applied onto the grid, followed by a drying period of three minutes. The coated grid underwent a drying process and was subsequently analyzed using a transmission electron microscope (Tecnai G20, Super twin, double tilt, FEI) operating at an acceleration voltage of 200 kilovolts [49].

### 3.5. Antibacterial Activity

The antimicrobial activity of thymol and nanoemulsified thymol was evaluated using the disc diffusion technique [50]. For antibacterial screening, *Bacillus cereus* ATTC 11778, *Staphylococcus aureus* ATCC 25923, *Escherichia coli* ATCC 35218, and *Klebsiella oxytoca* ATCC 51983 were used. The minimum inhibitory concentration of 50% was calculated as the lowest concentration of thymol or nanoformulation that inhibited 50% of growth. Growth dynamics were measured spectrophotometrically (at an optical density of 600 nm) every hour for 24 h. Cell suspensions (100 μL) were incubated in the presence of thymol or the nanoformulation in 96-well plates. Mueller–Hinton agar (MHA) plates were coated with 100 µL of each strain at a McFarland turbidity of 0.5. Sterilized 6 mm filter papers loaded with 50 µL of thymol and/or nanoformulated thymol were aseptically placed onto the middle of the plates, and the plates were incubated at 37 °C for 24 h. After the incubation, a measurement tool was used to determine the radius of the inhibitory zone. By employing the micro-broth dilution technique within resazurin dye, the 50% inhibition concentration (in micrograms per milliliter) (MIC50) of thymol or its nanoformulated form containing the same quantity of thymol against *Bacillus cereus* ATTC 11778, *Staphylococcus aureus* ATCC 25923, *Escherichia coli* ATCC 35218, and *Klebsiella oxytoca* ATCC 51983 was determined [51]. Thyme essential oil (25 g) was dissolved in dimethyl sulfoxide (25 mL), and the volume was made to 25 mL with sterile MHB containing 1% Tween 80 to provide a stock solution containing 500 mg/mL of oil. Plates were incubated at 37 °C overnight with various doses of thymol or its nanoformulated form that included the same amount of thymol (500, 250, 125, 62.5, 31.25, 15.62, 7.81, and 3.90 mg/mL). The optical density of every well was measured using a microplate reader (at 600 nm after 24 h of incubation, the difference in optical densities of each sample was compared, and MIC50 values were estimated). By subculturing the examined organisms, especially those with wells that followed those MIC50 amounts onto plates containing MHA at 37 °C for 24 h, the minimum bactericidal concentration (MBC) was also ascertained. Then, MBC was found for the nanoformulated of thymol that inhibited the development of tested bacteria at the lowest concentration [52]. At the chosen time points, the practical bacterial number was measured utilizing resazurin dye. The colonies were checked after 24 h of incubation. The time taken to kill the starting bacterial loads was evaluated by plotting the log CFU/mL versus incubation time.

### 3.6. Antifungal Activity

Antifungal activity of the Th-emulsion and Th-nanoemulsion was assessed against *Candida albicans* ATCC 90028, *Aspergillus brasiliensis* ATCC 16404, and *A. fumigatus* ATCC 204305 using the agar well diffusion method [53]. The fungal suspension, containing a concentration of 10^7^ spores per milliliter, was evenly dispersed across PDA Petri dishes. A septic cork borer with a diameter of 7 mm was employed to create a well in the plates that had been previously inoculated. Subsequently, 100 µL of the Th-emulsion, Th-nanoemulsion, and reference antifungal, specifically nystatin, was introduced into the well. All PDA plates were incubated at 30 °C for 72 h, and then the inhibition zone diameter was measured. The MIC of the Th-emulsion and Th-nanoemulsion were analyzed using the broth microdilution method [54]. The broth microdilution method was applied to detect the MIC of all tested fungal stains. Briefly, in a microplate, 10 µL of each fungal strain was added to a Sabouraud Dextrose broth amended with different concentrations of the Th-emulsion and Th-nanoemulsion (100–0.19 mg/mL), then incubated at 30 °C for 48 h. To determine the MIC for unicellular fungi, 20 µL of resazurin dye was added. A visual assessment was done for dye turning frm blue to pink inside viable cells. On the other hand, the MIC for filamentous fungi was detected by examining growth visually without adding dye. Then, the MFC was determined for all fungal strains; 10 μL of the clear or invisible growth wells were transferred onto SDA plates (10 μL/plate). The SDA plates were checked after incubation for 72 h at 30 °C [55].

### 3.7. Anti-Proliferative Activity

Employing the standard MTT method, the in vitro anticancer effects of Taxol, Th-emulsion, and Th-nanoemulsion were assessed against a panel of two human cancer cell lines, MCF-7 and HepG2. A positive control was used, which was Taxol. Half-maximal inhibitory concentration (IC50) values for all substances were used to express the results. Living cells can transform the yellow product MTT into the blue product formazan using a reduction reaction that occurs in the mitochondria. In this experiment, 5000 cells per well were placed in a 96-well plate, given 24 h to develop, and then given 48 h of exposure to a medium containing various concentrations of the test substances (0, 0.1, 1, 10, 100, and 1000 µM). Each experiment was conducted in triplicate. After the media had been removed from each well, 100 µL of MTT was added to each well, and each well was incubated for 4 h. The generated formazan product was solubilized with 100 µL of DMSO before being measured using an ELISA microplate reader (Epoc-2 C micro-plate reader, Bio Tek, Winooski, VT, USA). The concentrations required to inhibit cell viability by 50% were calculated using the calculated IC50 values.

### 3.8. Evaluation of Caspase-8 and Caspase-9 Activities

For the evaluation of casp-8 and casp-9, respectively, casp-8 (human, EIA-4863) and casp-9 (human, EIA-4860) ELISA kits (DRG International Inc., Springfield, NJ, USA) were utilized. The MCF-7 cell line was used to study the effects of Taxol, Th-emulsion, and Th-nanoemulsion on casp-8 and casp-9. Following treatment of the MCF-7 cell line with the previous compounds, the cells in the culture medium were incubated for 48 h at 37 °C in a humidified 5% CO2 atmosphere. The supernatant was removed from the cells, which were then washed once with phosphate-buffered saline (PBS) and harvested by scraping and gentle centrifugation, and PBS was aspirated, leaving an intact cell pellet. The pellet was resuspended in a 1X Lysis Buffer, incubated for 60 min at room temperature with gentle shaking, and the extracts were transfered to microcentrifuge tubes and centrifuged at 1000× *g* for 15 min. The activities of both casp-8 (pg/mL) and casp-9 (pg/mL) were measured in the samples using an ELISA kit [56].

### 3.9. Assessment of the VEGFR-2 Concentration

To quantify the in vitro concentration of VEGFR-2 following the manufacturer’s instructions, an Enzyme-Linked Immunosorbent Assay (ELISA) kit (Cat. No. EK0544) from AVIVA System Biology, USA was used. The MCF-7 cell line was used to study the effects of Taxol, Th-emulsion, and Th-nanoemulsion for their ability to inhibit VEGFR-2. Following treatment of the MCF-7 cell line with the previous compounds, the cells in the culture medium were incubated for 48 h at 37 °C in a humidified 5% CO2 atmosphere. After the cells were collected, they were homogenized with saline using a tight pestle homogenizer until all of the cells were broken up. The kit measured the amount of human VEGFR-2 in the samples using an ELISA that was sandwiched between two antibodies. Onto 96-well plates, an antibody specific to VEGFR-2 was precoated. Next, a VEGFR-2-specific antibody conjugated with horseradish peroxidase was added, and the mixture was incubated. Unbound conjugates disappeared in the wash. The HRP enzymatic reaction was observed using the TMB substrate. HRP catalyzed TMB to form a blue product, which became yellow with the addition of the stop solution. Yellow density was correlated with human VEGFR-2 levels. At 450 nm wavelength, the optical density was measured spectrophotometrically. After triplicate measurements, the concentration of VEGFR-2 in the samples was estimated (ng/mL) [57].

### 3.10. Statistical Analysis

All of the results were evaluated using GraphPad Prism 8.0 (2019, San Diego, CA, USA). The data were reported as the mean ± SD and emerged from at least three separate investigations (n = 3). The significant differences between the results of each group were investigated using ANOVA and Tukey’s multiple comparison tests. *p* < 0.05 was regarded as significant.

## 4. Conclusions

In this study, a Th-nanoemulsion was prepared from thyme essential oil and characterized using DLS and TEM analyses. The result revealed that the prepared Th-nanoemulsion had a spherical shape with a size of 20–55.2 nm. Furthermore, Th-nanoemulsion was assessed for antibacterial, antifungal, and anticancer activities. The micro-broth dilution experiment was used to evaluate Th-emulsion and Th-nanoemulsion in vitro antibacterial activity. In this regard, the MIC50 and MBC values for thymol were about 3–4-fold higher than those of the thymol nanoemulsion, showing that the Th-nanoemulsion was more efficient in preventing the growth of the tested organisms. Furthermore, the Th-nanoemulsion displayed promising antifungal activity against filamentous fungi (*A. brasiliensis* and *A. fumigatus*) but weak antifungal activity toward unicellular fungi (*C. albicans*). Additionally, the anticancer effects of Taxol, Th-emulsion, and Th-nanoemulsion were assessed against MCF-7 and HepG2 cell lines. MCF-7 breast cancer cells had the lowest IC_50_ values, indicating the most effective cytotoxic impact. Also, the Th-nanoemulsion resulted in the lowest IC_50_ value. Moreover, treatment of MCF-7 cells with the Th-nanoemulsion achieved the highest increase in the activities of both casp-8 and casp-9. The Th-nanoemulsion treatment also gave a lower level of VEGFR-2. Consequently, the Th-nanoemulsion has a potential anticancer effect as it reduces the growth and proliferation of HepG2 and MCF-7 cancer cells, increases apoptosis by increasing casp-8 and casp-9 activities, and finally lowers the level of VEGFR-2 concentrations.

## Figures and Tables

**Table 1:** Inhibition zones and MIC50; 50% inhibition concentration of thymol emulsion and thymol nanoemulsion and ciprofloxacin.

	*K. oxytoca*		*E. coli*		*B. subtilis*		*S. aureus*	
	IZ/mm	MIC50 (mg/mL)	IZ/mm	MIC50 (mg/mL)	IZ/mm	MIC50 (mg/mL)	IZ/mm	MIC50
Thymol emulsion	14 ± 2.1	ND	19 ± 1.8	ND	18 ± 1.8	ND	16 ± 1.8	ND
Thymol nanoemulsion	21 ± 1.9	62.5	23 ± 2.4	62.5	25 ± 3.9	250	26 ± 2.8	125
Ciprofloxacin	14 ± 3.2	50	16 ± 29	100	22 ± 29	50	21 ± 29	50

**Table 2:** Inhibition zones and MICs of the Th-nanoemulsion and Th-emulsion toward the tested fungal strains.

Fungal Strain Used	Inhibition Zone/mm			MIC/MFC (mg/mL)	
	Th-Emulsion	Th-Nanoemulsion	NS	Th-Emulsion	Th-Nanoemulsion
*A.* *brasiliensis*	13.2 ± 1.41	20.5 ± 1.32	10.2 ± 1.22	50/100	12.5/50
*A. fumigatus*	18.3 ± 1.1	26.4 ± 1.34	9.6 ± 0.57	12.5/50	6.25/12.5
*C. albicans*	12.7 ± 0.88	12.0 ± 0.90	ND	50/100	50/100

## Data Availability

Not applicable.

## Sample Availability

Not available.
